# Acutely admitted medical patients have increasing one-year mortality with increasing age

**DOI:** 10.1186/1757-7241-23-S1-A36

**Published:** 2015-07-16

**Authors:** Mikkel Brabrand, Daniel P Henriksen

**Affiliations:** 1 Emergency Department, Sydvestjysk Sygehus, Esbjerg, Denmark; 2 Emergency Department, OUH Odense University Hospital, Odense, Denmark; 3 Institute of Regional Health Research, University of Southern Denmark, Odense, Denmark; 4 Department of Clinical Chemistry and Pharmacology, Odense University Hospital, Odense, Denmark

## Background

The majority of patients admitted to a medical admission unit are old. Previous international studies have shown increased one-year mortality with increasing age. We performed the present study to clarify if the one-year mortality of Danish medical patients increased with increasing age.

## Methods

We followed a cohort of all consecutive acutely admitted adult (15+ years) medical patients for one year after admission. The patients were admitted from October 2008 through February 2009 and February through May 2010 to the medical admission unit at Sydvestjysk Sygehus, Esbjerg. Only patients who were not Danish residents were excluded. We extracted survival status from the Danish Civil Register and calculated mortality at one year stratified by age in the groups 15-49, 50-64, 65-79 and 80+ years. We calculated both crude hazard ratios using Cox Proportional Hazard Regression analysis and adjusted for potential confounders: gender, comorbidity (using Charlson comorbidity index [CCI]) and the British national early warning score (NEWS) at time of admission.

## Results

A total of 5,894 patients were admitted, 36 were not Danish residents and were excluded, leaving 5,858 patients for analysis. Median age was 65 (range 15-107) years, 2,923 (49.9%) were female, and 1,759 (30.0%) presented with severe comorbidity (CCI > 2). The crude one-year all-cause mortality was 18.6% (95% Confidence Interval [CI]: 17.6-19.6). The one-year mortality of patients 15-49 years was 2.5% (95% CI: 1.8-3.5), 50-64 years 13.3% (95% CI: 11.6-15.2), 65-79 years 23.8% (95% CI: 21.9-25.8) and 80+ years 37.2% (95% CI: 34.5-40.1). Crude hazard ratio for patients 50-64 years was 5.7 (95% CI: 4.0-8.1), 65-79 years 10.7 (95% CI: 7.6-15.1) and 80+ years 18.2 (13.0-25.6) compared to patients 15-49 years old. Adjusting for gender, CCI and NEWS, hazard ratios for patients 50-64 years old was 4.9 (95% CI: 3.1-7.6), 65-79 years 7.3 (95% CI: 4.7-11.1) and 80+ years 12.6 (95% CI: 8.2-19.4) compared to patients 15-49 years old.

## Conclusions

One-year mortality for acutely admitted medical patients increased almost linearly with increasing age. Even patients aged 50-64 years had a significantly increased one-year mortality compared to patients 15-49 years old.

